# Teachers as first responders: classroom experiences and mental health training needs of Australian schoolteachers

**DOI:** 10.1186/s12889-023-17599-z

**Published:** 2024-01-23

**Authors:** Harshi Gunawardena, Rose Leontini, Sham Nair, Shane Cross, Ian Hickie

**Affiliations:** 1 University of Sydney, Sydney, Australia; 2 The University of New South Wales, Kensington, Australia; 3 Department of Education, New South Wales, Australia; 4 Orygen, Parkville, Victoria, Australia

**Keywords:** Child/adolescent mental health, Curriculum, Teacher training, School, Australia

## Abstract

**Background:**

Schoolteachers are often the first to respond when a student presents with a mental health issue in the classroom. This places a burden on schools that impacts school staff, healthcare workers and teachers. More broadly, it places a responsibility on the education system to address students’ mental health. This study examines Australian teachers’ classroom experiences and the training areas identified by teachers as necessary to manage these issues.

**Method:**

Interviews were undertaken with 18 in-service teachers between 2020 and 2021 from Catholic, Independent and Public schools. Data were gathered via multiple interviews and analysed using thematic content analysis.

**Results:**

The major mental health issues identified by teachers related to mental disorders, depression, anxiety, and a complex range of negative emotional states. Teachers requested training in child and adolescent mental health, counselling skills, early detection and intervention, and training skills to manage the complex relationship with parents and external health and community personnel. Teachers also reported the need to access mental health resources, support and training, which were differentially accessed along socioeconomic status and postcodes.

**Conclusion:**

The data show that teachers are often placed as first responders when a student has a mental health issue but feel inadequately trained to manage these issues in the classroom. We identified mental health issues presenting in Australian classrooms and documented critical features of mental-health training asked for by teachers in order to address those issues. Given the increasing demands on teachers to address the mental health of children and adolescents, we argue that an urgent review of mental health training for teachers is needed.

## Introduction

Global trends indicate that almost a quarter of adolescents will experience a mental health issue in any given year [1]. Mental health issues can present in school settings as temporary negative emotional states, a non-specific mix of subthreshold disorders, to full-blown disorders. Key national studies in Australia show that 26% of youth reported a mental health issue in the last 12 months [2, 3]. These issues include attention deficit and hyperactivity disorder (ADHD) (8.6%), anxiety disorders (7.0%), and oppositional problem behaviours (5.3%) [4]. The Australian government’s response to child and adolescent mental health is exemplified in national reforms, namely the *National Safe Schools Framework* (2011) [5], the *National Review of Mental Health Programmes and Services* (2014) [6], and the NSW *Well-being Framework* (2015) [7]. In addition to Medicare based government funding for psychological care, the Australian government supports a range of programs including *Emerging Minds*, *Better Access, Be You*, *Headspace, KidsMatter* and *MindMatters*. Despite these initiatives, the *Productivity Commission Report* found:

“The Australian Government’s …. initiatives do not address the fundamental issues that impede schools from making a measurable difference to mental health and wellbeing, including: … inconsistent approaches to teacher pre-service training and professional development in mental health and wellbeing” [8] (p. 216).

Mental health training requirements in initial teacher training (ITT) and in-service training across states and territories are inconsistent. As a result, mental health outcomes are not compulsory in the national reporting framework’s outcomes, targets or measures under the *National School Reform Agreement* [9]. This creates a dilemma for teachers in that they recognise that supporting students’ mental health is part of their role [10], yet the training required to support their mental health is inadequate or inconsistent [11–13]. The World Health Organisation (WHO) defines mental health as:

A state of wellbeing in which the individual realises his or her own abilities, can cope with the normal stresses of life, can work productively and fruitfully, and is able to make a contribution to his or her community [14].

This broad definition of mental health is particularly relevant to schools that are also invested in helping individuals realise their abilities through education. Mental health issues can adversely impact a child or adolescent’s education leading to disengagement with school, employment, or training (NEET) [15–17]. Teachers are often the first professionals to observe a child having a mental health episode. Teachers are then compelled to initiate responsive action via school protocols that initially involve school personnel [18–20].

In a paper titled ‘The Legal Definitions of “First Responders”’, a definition of this term is given as: “those who would be the first to respond in the case of an … emergency” [21 (page 1)]. Costa and colleagues adapted this definition to teachers who fall into the category of ‘atypical professionals’ required to provide a response in the event of a crisis [22]. Beames and colleagues suggest that teachers fulfil this role insofar as they are the first point of contact when a child or adolescent faces a mental health crisis requiring the access to mental health services [23]. Teachers then engage instigate a triage system where the school contacts mental health specialists and implements schools-based programs and strategies of care. According to Australian government health and education policy [24], teachers’ roles are defined in terms of Tier 1, 2 or 3 interventions. Within the school context, Tier 1 interventions involve universal prevention processes; Tier 2 involve targeted interventions with small groups or individual students who have been diagnosed with a mental health condition or are at risk of developing a mental health problem; Tier 3 interventions involve intensive individualised support. However, the question remains whether Australian teachers have been adequately trained to manage these levels of mental health care needed to support students.

Over the past decade, teachers have been calling for more training [25–27] which may be tied to two contributing social factors: an increase in the diagnosis of mental health issues among children and adolescents [28] and inequities in the level of resources of mental health support across schools located in different postcode areas [29–31], leading to what researchers describe as a widespread sense of helplessness among teachers [32–34]. However, research on teacher experiences has, for some time, documented the inadequacies of teacher training to address students’ mental health issues [35–37]. Internationally, teachers in similar developed countries seemed to face similar training issues: Shelamy and colleagues, who conducted interviews with seven UK teachers, described a level of frustration among teachers who believed that their education programs did not provide sufficient preparation on child and adolescent mental health and did not equip them with the necessary skills to access relevant resources [38].

A qualitative study of 24 Australian and Scottish school teachers argued for increased mental health literacy in teacher education in both of these English-speaking countries, on the grounds that teachers can respond more effectively to students’ mental health issues if more training is provided [39]. Previous research of 100 final-year trainee teachers in Australia used vignettes to present case scenarios for trainees to assess [40]. The mental health curriculum that the trainee-teachers were taught in the study was described by the trainee-teachers as “unclear”, leading the authors to recommend that a review of training curricula is needed in Australia. Indeed, a qualitative study of 52 participants in the UK found that teachers and other school staff also self-reported that they felt they lacked mental health knowledge to manage the mental health issues of students in their classrooms [41].

Research suggests that the lack of mental health content in teacher training curricula can be helped by providing more training. For example, an Australian study of schoolteachers found that teachers’ knowledge and self-efficacy in managing students’ mental health issues increased significantly with training from the KidsMatter teacher-training program [42]. While research shows that training in mental health increases teachers’ confidence and knowledge of health issues [43, 44], the inadequacies in training continue to be voiced by teachers. Teachers continue to ask for training that tells them: “here is what to do, or here is what to say” when a child or adolescent is experiencing a mental health issue [45]. Research suggests that studies need to address whether and how the perceived gap in teacher training is being addressed [41]. For example, in a US survey of 786 educators, 85% stated that they needed further training in mental health, specifically in mental health disorders, behaviour management, and social and emotional skills training [46]. Other studies in the US found that training in suicide prevention was also important for youth mental health [47]. A UK study reported that teachers asked for more collaboration between schools and community-based mental health professionals and mental health services [41]. Overall, the research from Australia and comparable English-speaking countries suggest inadequate training is a critical barrier to providing mental health support to students in their classrooms [26, 46, 48].

The inadequacies of teacher training programs have become particularly critical for low-SES schools. The German BELLA cohort study of 2,111 students suggested that mental health issues are more prevalent in children and adolescents from low socioeconomic status (SES) backgrounds [49], while a UK survey of 23,215 children found that lower SES of school location correlated with higher behavioural and mental health issues [50]. In Australia, not all schools are sufficiently resourced to assist students and/or teachers in managing students’ mental health. Similar to other English-speaking countries, schools located in areas of socioeconomic disadvantage reported higher cases of student mental health issues (Anon, 2020). The findings from previous research suggest increasing reliance on teachers to act as first responders when students present with a mental health issue. The present paper builds on this research by providing more nuanced consideration of the development of curriculum topics for teacher training in child and adolescent mental health. More specifically, we report on Australian teachers’ experiences with child and adolescent mental health issues and what teachers believe are essential but insufficiently covered topics in current teacher training in mental health.

In light of increasing mental health diagnoses in schools and the inadequate preparation to meet increasing demand, the aim of this paper is to inform future teacher training programs in Australia to better meet the needs of children and adolescents experiencing mental health issues in school.

## Method

This paper reports the findings from 18 interviews with current serving schoolteachers in Australia. Ethics approval was obtained from the University of Sydney Human Research Ethics Committee and the NSW State Education Research Applications Process.

### Participants

All participants were teachers in Australia’s public public, private and independent schools, both in rural and greater Sydney metropolitan areas. The teaching experience ranged from 3 to 30 years; the average was 16 years (Table 1).

**Table 1:** Participant characteristics

	N = 18
Gender	
Male	6
Female	12
Years of teaching	
0–5	3
6–10	3
11–15	2
16–20	5
21–25	2
26–30	3
School Level	
Primary	6
Secondary	12
School Gender Category	
Girls	1
Boys	1
Co-Educational	16
School Type	
Public	13
Private	2
Independent	3
Local Government Area	
Bayside	2
Fairfield	1
Hills	1
Inner West	5
Liverpool	2
Narrabri	1
Newcastle	1
North Sydney	1
Northern Beaches	1
Parramatta	1
Sydney	2

Recruitment for this study occurred through multiple sources: online advertisements posted on social media (Facebook teachers’ groups); brochures distributed at schools and education department office noticeboards; letters and telephone communication with school Principals; and alumni groups (from the education faculties at universities) between April 2020 and April 2021. Participants met the criteria for inclusion if they were current serving teachers. Interviews were conducted by the first author at the university campus, online via Zoom, Facetime, or telephone; other choices were also offered, including their school and a café. Before the interview commenced, privacy, confidentiality and consent procedures were explained and followed in accordance with HREC requirements. Therefore, all participants’ names were replaced with pseudonyms and any data that can identify them were removed.

### Data collection

The data were collected through 18 semi-structured interviews conducted by the first author. The total duration of interviews ranged between 30 and 90 min. Before the interview, sociodemographic information was collected, including years of service, teaching history, teaching area, and postcode area of the school. Follow-up interviews were conducted with some participants to mine deeper into specific emergent topics raised in their interview. Respondent validation was applied to the transcribed data to check that the participants’ views were reported correctly [51]. Interviews focussed on teachers’ experiences of students’ mental health issues in the classroom. To address these key areas of investigation, we developed three open-ended questions in the interview schedule, which were provided to all interviewees prior to the interview. Examples of the type of questions included: ‘Can you please tell me about any particular incidents that you have experienced in the classroom that you believe are related to students’ mental health?’, ‘What training (initial teacher training) or professional development were you given in order to handle such situations?’

### Data analysis

All interviews were manually transcribed verbatim immediately after the interview or follow-up interview. The purpose of the research was to identify teachers’ experiences managing the mental health issues of students in their classroom. The data were captured in Microsoft excel and reported using ‘*Standards for Reporting Qualitative Research*’ [52], and analysed using thematic content analysis as outlined by Bruan and Clarke [53–55].

First, a deductive process of manifest surface coding of transcripts was conducted. This process was based on quantitative counts (frequency) of key words (codes) within qualitative data (interview transcripts). As described by Morgan and colleagues [56], this type of coding involves counting the number of times a code appeared in a transcript, and is a method found in previous mixed method and interdisciplinary research [57–59].

The first process was deductive and was informed by a coding frame. The items in the first coding frame were mental health issues in the classroom and were based on the 5-item list of mental health issues reported in the Australian landmark child and adolescent survey by Lawrence and colleagues [4]. The result of this coding process is reported in tabular form in the section ‘*Students’ mental health issues*’.

Following deductive coding, the coding process turned to inductive ‘open coding’ [60] of the qualitative data, which was analysed using manual thematic content analysis following Braun and Clarke [54]. This process offered a flexible yet structured approach to finding ‘patterns’ in the transcripts. Keywords were identified in the transcript and similar codes were clustered together to form themes. The codes were organized in an excel sheet, and each code in the excel sheet was accompanied by direct quotes from (anonymised) participants. For example, the individual codes: classroom management, behavioural issues, anger management, restraining students, calling the police, ‘one student who always fights’, ADHD as well as other codes, formed the sub-theme ‘*Classroom behaviour*’. Six major themes related to mental health issues in the classroom are presented in this paper.

Data were analysed until saturation. However, saturation was not simply achieved through a repetition of keywords or codes appearing in subsequent transcripts; rather, as Neilson [61] and Anderson [62] have argued, saturation is met when the same keywords or word clusters no longer produce new perspectives on the same theory in successive transcripts. However, in qualitative research we acknowledge that new perspectives will always be possible and therefore see this as a limitation of the present study, but also as a basis for future research that identifies more complex mental health issues in schools.

## Results

Eighteen currently serving schoolteachers participated in the research. They taught in public, private and independent schools in rural and greater Sydney metropolitan areas. The teaching experience ranged from 3 to 30 years; the average was 16 years (Table 1).

### Students’ mental health issues in the classroom

Teachers in this study reported that the most common mental health issues presenting in their classrooms were anxiety, depression, ADHD, and behavioural issues. The kind of mental health issues and the number of times that each mental health issue was mentioned by each teacher in our study are collated in Table 2.

**Table 2:** Mental health issues presented in the classroom

Mental health issues	Number of teachers who reported the issue N = 18		Total %
Anxiety	17		94%
Depression	12		67%
Attention deficit/hyperactivity disorder (ADHD)		10	56%
Behavioural issues	10		56%
Autism spectrum disorder (ASD)	6		33%
Self-harm	6		33%
Suicidal tendencies	5		28%
Negative self-image	5		28%
Eating disorder	4		22%
Identity issues	3		17%
Bipolar	2		11%
Obsessive-compulsive disorder (OCD)	1		6%
Schizophrenia	1		6%

These conditions were diagnosed by a healthcare professional and registered in the schools’ centralised ‘Learning Management System’. However, teachers often had to identify the nature of the mental health condition and find immediate strategies for classroom management for the student and their peers. As discussed below, some students came to school with undiagnosed mental health conditions.

#### Depression and anxiety

Participating teachers identified anxiety as the most common mental health issue presenting in the classroom, “I have students with anxiety, anxious children, fragile little girls, change of life babies with overprotective parents” (Jean). Jean was an experienced teacher at a public school in a low SES area who explained that one of her students was “constantly stressed and anxious”, and while she suspected he was not receiving appropriate care, she also felt helpless: “I didn’t have sufficient knowledge and skills to help him”. Maxwell, who teaches in a medium to high SES school located in the metropolitan centre, explained that anxiety is the most common mental health condition he found among his students. However, in his experience, episodes were mostly related to school-based factors such as “academic issues, sporting challenges, or friendship break-ups”. Two thirds of teachers in this study reported students with depression in their classrooms. An experienced teacher with 18 years of teaching said, “I’ve seen a lot of students with depression” (Joanne). However, depression is often comorbid with other mental health issues. Teachers encountered students with multiple issues: “I had one student, he had depression, anxiety, ADHD, ASD - a lot of issues for a young man to manage” (Ethan). When students present with multiple mental health issues, the demands placed on teachers to manage the student and the other students in the classroom increases considerably.

#### Classroom behaviour

Gretta, an experienced teacher in a public school in a low SES area, indicated that addressing mental health issues is deferred because priority is given to behavioural management: “My school has spent the whole year on classroom management”. Gretta articulated her school’s experience as follows:

We have one school psychologist who comes one day a week. The closest paediatrician is 200 km away. We’ve had two violent lockdowns this year for one kid because the police are 40 min away! (Gretta)

Structural factors such as location and poor resourcing can create challenges for teachers in managing students’ mental health issues in schools. In Gretta’s case, such factors included limited access to school counselling services (psychologists) and specialist healthcare workers. Being in a rural area, there was poor access to specialised services. However, while location isolation was a critical factor in reducing her capacity to refer students to specialist support, some teachers in metropolitan low SES schools also expressed similar concerns accessing mental health services.

By contrast, teachers from well-funded private and selective schools in high SES areas described very different experiences in relation to behavioural management. Samantha, who teaches in a selective school said: “Basically, we have no behavioural issues in the classroom”, adding that students in her school all wanted to be there and to do well in their studies.

Unlike other mental health issues, behavioural issues raise unique challenges to teachers because they are faced with trying to understand whether behavioural issues are due to a child’s frustration with learning problems or if they are due to mental health problems:

It could be that they have low literacy and numeracy skills, which could transpire as behavioural issues… As a teacher you need to know the difference (Millie).

From teachers’ accounts, it emerged that the term ‘behavioural issues’ is used as an umbrella term to describe one of two possibilities: either an aggressive response due to being frustrated with learning problems, or as undiagnosed mental health issues transpiring as behavioural problems. Through academic assessment tasks, teachers can learn about and support learning difficulties: however, teachers are challenged when understanding the nature and cause of students’ mental health. For example, appearing as behavioural issues in the classroom were also cases of anger management and ADHD, ASD, self-harm, as well as students with suicidal tendencies (see Table 2):

Anger management, attention deficit disorders, and suicidal tendencies. I had to restrain an autistic student who had a meltdown the other day. Others self-harm or harm someone else, or have oppositional defiance disorder… Teachers need to know how to identify these. (Joanne)

As this excerpt shows, in addition to one student presenting with multiple mental health issues, a classroom can include students who present with a number of different mental health issues. Our data show that teachers reported having sufficient behavioural management knowledge and skills learned through their teacher training and professional development, however, that such learning was different to what is needed for mental health training. This difference is illustrated by Ethan in his encounter with one student who had multiple problems:

I had one student … I started teaching the topic we were working on the day before, but then something set him off straightaway. He went from nought to hundred within five seconds. He threw a pen at one student, then he threw the desk over… I thought wow, what happened?

Prior to entering the classroom, Ethan had read the learning needs and background information related to the student via the centralised learning management system. Yet this information was insufficient to prepare Ethan for the student’s sudden outburst, leaving Ethan in consternation. As Ethan’s experience illustrates, there is a need to identify the difference between a violent outburst that is driven by a learning problem or a mental health problem, because if it is the latter, then the response to the student has to be delivered in a different way. Ethan explained that the student would normally be punished for such an outburst by being sent to the principal’s office: however, punishing a student is not the solution when that student has a mental health issue. While mental health issues of students are escalated via the school system, Ethan asked for training to know what to do at that first instant that a student experiences an episode or presents with a mental health issue in the classroom.

### Teachers’ role as first responders

While there are strategies in place to manage the mental health and wellbeing of children in schools through escalating levels of care, the instant a child experiences a mental health episode in the classroom, the teacher is the first adult to interact with that child. This position places them in a crucial role:

The classroom teacher serves a crucial role because it is the classroom teacher who alerts everyone to the problem: … [that] this child is not behaving normally. The classroom teacher … is the one who first picks up the issue. (Millie)

When asked how one teacher first noticed that her student had a mental health issue, she said: “The marks were affected, that’s how I first picked it up” (Samantha). Students’ marks are a unique tool available to teachers. Changes in academic outcomes offer teachers insight into psychological changes that their students may be experiencing. In these situations, teachers find themselves as first respondents:

A lot of teachers are put in a situation where you become the first responder. So, teachers need the training to know what to recognise … [and] the warning signs. (Casey)

Another teacher, Denzel, explained that he was the first person to notice the deteriorating mental health condition of one year-12 student in his classroom when he noticed that the student’s “replies were odd”. When the parents were contacted, they said that they thought something was wrong with their child but did not know what to do about it. Denzel was then able to escalated the matter through the school system to provide the mental health care that the child needed.

While the core business of schools is education, teachers like Denzel are placed in situations where they can see that a student is experiencing a mental health issue and then are required to instigate appropriate care. Teachers expressed frustration at not being able to understand what a child is experiencing at that first point of contact, even when they could see that something was wrong. Some teachers said, “I want to help” (Samantha), while at the same time, other teachers said that they did not know what to do: “what can I do?” (Ethan). This dilemma led many teachers to what was described by one teacher as a: “feeling of powerlessness” (Hayley) where they found themselves in the role of a first responder; but lacking the minimum skills in mental health training to respond as a first responder.

### Accessing school counselling services

The first point of call when a student experiences a mental health issue is the school counsellor. They are largely responsible for the referral service required to escalate care. Teachers in our study described different experiences accessing counsellors for students, which were affected by school socio-economic postcodes. For example, Jean, who teaches in a public school in a low SES metropolitan area, said this about the school counsellor:

What counsellor? We get a new one every year. The last counsellor moved on after two or three months. We got a brand-new counsellor, first year out [of university]. Two counsellors have 5 or 6 schools. So, they spend one and a half days per school. (Jean)

Due to the high demand for counsellors and the low number of counsellors available for public schools, Clive summed up his experience as follows: “access to counsellors is limited. There’s not really an opportunity to have an effective discussion with them, that is quite scarce”. Ainsley, who teaches in a public school in a low SES area explained that there are long waiting times at his school to see a counsellor: “it takes something like a month to see the counsellor. This is a really big problem”.

Given the shortage of school counsellors in Australia [29], teachers in this study often found themselves performing the counsellor’s duties, despite not having the appropriate training or qualifications: “I ended up having to take the role of the counsellor” (Hayley), and Maxwell said: “the principal took on the role of the counsellor” in his school. The lack of access to school counsellors resulted in school teachers using other personnel to fill the gap, including pastoral care services (Susila). Casey explained that in his school they used an unregistered social worker to help with the lack of school counsellors needed for their students. Alternatively, the school could obtain an external psychologist to support a particular student. However, in Joanne’s experience, she explained that external psychologists do not always understand that “what is ideal for a particular student is not necessarily what is feasible within the school environment” (Joanne).

The experiences of school teachers in this study suggests strongly that training in school counselling is needed for all teachers at initial training and as professional development. However, access to that training also varied across socio-economic postcodes. Thomas teaches in a school located in a high SES area and he had access to professional developed:

I did Rocky Biasi’s professional learning workshops and Accidental Counsellor 1 and 2. But we need these for all teachers in our initial teacher training, not as PD for some and not others.

By contrast, Jean said that in her experience, courses in mental health are difficult to access. She said:

‘Accidental Counsellor’ – I couldn’t get onto it. I would jump through hoops if I could’ve got onto that because I want to know more, I want to help.

Jean’s comment highlights the need for a new approach to teacher training in mental health.

### Early detection and intervention

Students can often present in school with a range of mental health issues within the health continuum. These largely involve temporary negative emotional states and a non-specific mix of subthreshold conditions characteristic of conditions at the low end of the continuum. Teachers in this study said that such students were often not referred to the school counsellor due to the high demand for counsellors. Teachers expressed concern, however, about such students where their mental health issues were not acknowledged and therefore untreated:

It is the ones who are quiet who are showing early signs of disengagement, not submitting their work, absenteeism, hiding, not present. They go under the radar. They are the ones I am worried (Millie).

Teachers in this study requested training in the early detection of mental health disorders. Teachers believed early detection and intervention are key to effective mental health support: “early intervention is important” (Hayley). They reported that a mental health curriculum would benefit from teachers being trained to have the skills and knowledge to recognise early signs of mental health issues in children and adolescents. Delaying diagnosis also delays administering care, which is a significant problem for a child’s well-being and future mental health.

This challenge is more difficult when pre-existing mental health issues have gone undiagnosed: “A lot of kids come to school …. they’ve got multiple mental health problems, all undiagnosed” (Gretta). Teachers explained that sometimes they find that they are the first adult to identify that a student is experiencing a mental health problem.

The implication for teachers is that it becomes incumbent upon them to act as first responders and to initiate mental health assessment and care, which occurs at the first-point of contact with the student. In order to perform this function effectively, there is a critical demand among teachers to increase their education and training in child and adolescent mental health.

### Feeling unsupported

In addition to the stress caused by the lack of mental health training, there are other concerns related to teachers work. Some teachers in this study indicated withholding support or advice in fear of reprisal:

You are afraid of offering support or giving advice. You are careful not to offend or do something to really fix the situation outside of procedure in case… it will come back and bite you, even if you know it will work. (Ethan)

Ethan explained that teachers need to overcome this fear: “Teachers just want to know if something does go wrong, that they are not going to be hung out to dry”. Ethan spoke of the constant fear of disciplinary action if they mishandled a situation or did not follow protocol. Some teachers put it more strongly: “We are not supported” (Jean). Other teachers spoke of situations where they had had no support in the past: “The child is always right. Teachers are always blamed for everything. We have no say in what goes on” (Josephine). Josephine’s words indicate the lack of faith in a school system to support her.

A similar point raised by another teacher related to the level of trust given to teachers. Hayley explained that if she reported a student with a mental health issue, she would like to be informed about that student’s progress:

If you are the first person to alert the school to a situation, then you expect to be told how that student is going. But we are left out of the equation. It’s like we are not trusted. But I think it is important to have the teacher’s involvement. (Hayley)

It may be the case that the teacher was not informed about the student’s progress because her training was insufficient to understand the mental health issue. Despite a process of escalating mental health care within the school system, teachers are placed on the frontline every day in their classrooms. It is for this crucial point of contact as a first responder that mental health training was requested by teachers.

### Managing parents

The involvement of parents in the management of the mental health of students was a key issue raised by participant teachers in this study. However, there were many facets to parental involvement in the mental health of students. In some cases, the problem was that parents did not want to know that their child had a mental health issue:

Often parents don’t want to face the issue [that their child has a mental health issue] because it’s too confronting for them. [But] if we don’t get the partnership with home, then the mental well-being of the child: it is very very hard to get anywhere with that. (Nancy)

On the other hand, parents can be the cause of a child’s mental health problems. Hayley described a tragic case of one of her students whose parents divorced when the student was 10 years of age and was living with the mother in housing commission [public housing]:

The mother came to our attention when she [the mother] had beaten her [the daughter] due to a bad school report. She [the daughter] came to me … and she was crying in my office.

This student was described as “a DOCS [Department of Community Services] case” and was in different temporary accommodation before she was finally diagnosed with schizophrenia after leaving school. Teachers related stories such as the above, which they observed happening over a period of years, but were unable to intercept the trajectory of decline of a young life.

On the other hand, teachers also discussed the challenges they faced in managing parents who wished to participate too much in a child’s life. These are parents that one teacher described as “helicopter parents” (Maxwell). This terminology is used to describe parents who, like helicopters, ‘hover overhead’ overseeing every aspect of their child’s life. Children of such parents are often academically high-achievers. However, as Nancy explained:

This is very demanding on us – there are parents whose children never fall, never fail, never do anything wrong…. And that is not a good thing. They want their kids to perform all the time. So, that becomes a mental health issue because they don’t develop the social and emotional skills to navigate in the real world. This is a major problem in terms of child mental health, and it is very very common.

Parents can subject the child to unrealistic academic goals while denying them a realistic understanding of failure and success. Managing the mental health impact on the child of such parenting also poses challenges to teachers. Within the broader context of school and community partnership to manage a child's mental health, teachers requested training to manage parents and to understand and manage the effects of parental intervention on a child's mental health, as well as the subsequent communication needed with family and community services.

## Discussion

Mental health issues impact school outcomes, resulting in reduced academic performance, failure to submit work, disengagement from class activities, and absenteeism. The interaction between psychological and academic functioning allows teachers to observe changes in a student’s mental and emotional states. This unique tool can be utilised more effectively by acknowledging the important role that teachers can play and by including them in supporting the mental health of children and adolescents.

To date, mental health training is not a compulsory unit of study as defined by Australian national teacher training standards, despite an increase in diagnosed mental health issues among children and adolescents [26] and inequities in the level of resources of mental health support across schools being aligned along postcode areas [27]. As a result, many teachers in this study felt that more teacher training should be considered to support teachers. Over the last decade, teachers have expressed their concerns that more training and support are needed to manage students’ mental health issues in their classrooms [25, 33, 38]. While research has only started to reveal the extent of the issues teachers face, there has been little documentation of teachers’ classroom experiences when a student presents with a mental health issue. Recent research has voiced teachers’ concerns, most succinctly articulated in a UK study as first, the need to identify mental health issues and second, the need to identify areas of challenges related to training in mental health [41]. In the present paper, we have responded to this call by developing an extensive list of mental health disorders in the classroom that expand on previous lists and identifying specific challenges directly related to mental health training.

The first section involved identifying the range of mental health issues, which was achieved from information about teachers’ classroom experiences. Consistent with population prevalence [3], the most common mental health issues were anxiety and depression. Over half of the teachers encountered students with behavioural issues, mainly ADHD, ASD, anger management, and oppositional defiance disorder. Other student issues included suicidal tendencies, self-harm, negative self-image, eating disorder, identity issues and suicidal tendencies. These areas of mental health were similar to those identified in other English-speaking countries as necessary knowledge for training purposes [51]. Although behavioural problems are significantly featured in initial teacher training, teachers claimed that current teacher training only approached behaviour management in terms of ‘discipline’ rather than understanding that some behaviours may be a symptom of a mental health problem. The important distinction between a student behaving badly and a student with a mental health problem can only be made given the appropriate mental health training. Teachers’ reports in the UK also noted that their initial teacher training provides methods for behavioural management [41]. Our data suggests that modification is needed to teacher training to understand that managing a mental health issue is different to managing student behaviour and that training is mental health is needed to understand the difference.

One of the main issues little documented in current research is the presenteeism of students with undiagnosed mental health conditions, which poses a significant challenge to teachers. For example, teachers reported that students in their classroom without a mental health diagnosis showed visible signs of mental health problems, such as behavioural issues, disengagement, not submitting classwork, crying, self-harming, and truancy. However, without parental or a healthcare professional’s verification, teachers were forced to continue to manage those issues in the classroom without support. Considering the challenges already identified in accessing school counsellors in some schools in Australia, teachers suggested that mental health training would help them to manage the immediate classroom situation until a diagnosis by a healthcare professional is accessed.

One of the key issues prominent in the interviews with teachers was related to school counselling. Australian teachers explained that they identified with the title ‘accidental counsellor’ because they found themselves in this role, despite not having the training of a healthcare worker. Teachers also acknowledged the danger that the lack of teacher training posed to students with mental health issues, calling for mental health teacher training to be made mandatory for all teachers.

The lack of school counsellors emerged as a key issue, particularly in rural schools, and public schools in low SES areas. Participants described situations in which teachers and other school (non-qualified) personnel, were taking on the role of the school counsellor due to the dire shortage in Australia of school counsellors [29]. There was also disparity in the access to training in mental health, and popular professional development, such as ‘Accidental Counsellor’ which were identified by teachers in our study as imperative for all teachers rather than a select few. The request for training was unanimous amongst teachers in our study, which supports research from Scotland [39] and the UK [20, 36], which also found teachers asking for psychology training because, even though teachers are not qualified to provide a diagnosis, “they can, nonetheless, identify a child ‘in need’” [20] (p. 1229).

Teachers also indicated their understanding of the importance of early detection and intervention in managing mental health. Supporting the work of Anderson-Butcher [32], we found that teachers are essential to the referral process within schools that is needed to deliver mental health to students. Additionally, our data showed that teachers take on the role as first responder at that crucial point at which the student is in a mental health crisis. As the first adult to respond to this crisis, teachers in this study asked for mental health training to better prepare them for this crucial interaction. Anderson-Butcher [32] also noted that teachers needed training to monitor a student’s progress once an initial diagnosis had been made. In delving deeper into this issue, we found that teachers felt that they had been left out of the process by not being informed of the student’s progress. Our data suggests that at least some teachers wished to be informed about a student’s progress and recovery process because it helped them better prepare for the time that the student returns to their classroom.

Amidst the crisis of mental health management in schools was also a sense of lacking confidence to intervene when a child is experiencing mental health issue due to lack of training and a feeling of being unsupported. This conflict resulted in teachers describing a sense of both powerlessness and frustration. We suggest, however, that if teachers were at least adequately trained then they might be more confident about how they manage the mental health issues of students in their classrooms.

Another crucial factor that emerged from our research is the role that parents play in managing a child’s mental health. Previous research has highlighted the importance of engaging community, specially parents: but not necessary described how this interaction between parents and teachers work [14, 41]. Our contribution is to provide that information. Teachers in our study described cases of neglectful or violent parents and also parents who were over-protective or imposed performance pressure on students, all of which contribute to creating or exacerbating a child’s mental health. Other teachers described situations where parents were in denial of a child’s mental health problem, thereby denying access to care. Teachers requested training in having those awkward conversations with parents about the type of mental health issue their child might have, next step for diagnosis, and prognosis, and none of these techniques (neither knowledge or skills) are currently provided to teachers in their training. This is yet another reason why mental health training is needed for teachers.

The difference between schools in high and low SES areas emerged as a critical factor in our research. Teachers in public schools in low SES and rural areas identified more challenging issues than teachers in private schools and selective schools in Australia. Teachers in private and selective schools in high SES areas also reported having more training and professional development opportunities in child and adolescent mental health. However, teachers in low SES public schools said they had limited access to mental health training, which was compounded by a reduced capacity to access external child mental health training providers, as well as time constraints brought on by a crowded curriculum. School teachers in some low SES areas explained that special-education students and mainstream students were placed together in one classroom and that most of the teachers’ time was spent in behavioural management. Differences in behavioural events emerged between teachers from low SES public schools and private or selective schools in high SES areas. Although significant in the present study, more research with a larger nationwide sample will help to add support to these findings. Disparities related to SES also emerged regarding access to school counselling. Schools in low SES areas may receive one counsellor for as little as half a day per week, resulting in minimal and inconsistent care for students. Indeed, the *Review of Counselling Services for Children and Young People in NSW Government Schools* found school counselling services were inadequate, aiming for a low benchmark of 1 counsellor per every 500 students [29]. Such low standards question the quality of counselling services that can be provided for public school students.

Unsurprisingly, many schoolteachers found themselves in the role of having to perform counsellor duties. However, without the psychology training that school counsellors receive, teachers expressed feelings of ‘fear’ that they might exacerbate a child’s mental health issue, resulting in some cases of the teacher withdrawing their contact with the child completely. This sentiment is articulated by studies of teachers in similar developing countries [20]. Teachers described a sense of powerlessness and inaction when faced with having to respond to the mental health needs of students because they did not have the correct knowledge and skills to react appropriately to a child or adolescent’s mental health episode or ongoing issue. Feelings of powerlessness can negatively affect teachers’ mental health, and our research coming from the perspective of mental health supports previous research based on teacher burnout [63], both of which suggest that teachers are at risk of developing traumatic stress and compassion fatigue. The frustration described by Australian teachers is similar to those documented by teachers in the UK and the US [30–32]. The data in our study, however, suggest that teachers’ experiences at the classroom level are more dire than previous research had suggested.

There are many implications for both initial teacher training and professional development arising from this study. First, we identified teachers’ experiences when they come face to face with students with mental health issues in their classrooms. Our research echoes the call from teachers in other developed countries to provide teacher training in mental health [20, 39, 41]. Our research and previous research suggest an absence of or uneven access to mental health training for teachers [64]. Yet, little research has identified deeper issues related to managing the mental health of students. The present study has sought to address this gap by putting forward significant issues related identifying mental health problems of children equivalent to first responders; needing more support from schools, particularly in terms of building teacher capacity and confidence through upskilling; accessing learning modules; accessing school counselling for students; and managing parents, which the present research identified as a crucial school-community mechanism necessary for the welfare of a child with a mental health issue. We have argued for the importance of training because teachers’ changing roles have now placed them as frontline staff as they are often the first to respond when a child experiences a mental health issue. Therefore, our research supports the argument by previous researchers that “with the necessary knowledge, understanding and support, teachers can function as the ‘frontline’ of identification, support and referral to other services” [65] (p. 10). Undoubtedly, there is a rise in the reporting of mental health difficulties among young people, which needs responsive changes to school practices [66]. Teacher training has undergone many changes to adjust to the current environment, however, the *Productivity Commission Report* stated that current mental health training “do not address the fundamental issues that impede schools from making a measurable difference to mental health and wellbeing” (p. 216).

Returning to the World Health Organisation’s definition of mental health [14], we suggest that poor mental health training limits the support schools can practically provide their students. The present study found that some form of professional development in mental health is available to teachers in all schools. However, the absence of compulsory and consistent mental health training across states and territories has resulted in only the partial uptake of training and inconsistent training curricula nationally. We argue for an approach involving the Council of Australian Governments (COAG) is needed with terms of reference and representation of teachers’ voices identifying their mental health training requirements. To that end, this paper provides an initial exploratory work that articulates teachers’ voices in terms of what teachers asked for in their training in the hope that it will help policymakers to advance teacher training in Australia.

### Limitations of the current study

Our findings indicate that students are presenting with a range of mental health issues previously not identified in the detail we have provided. We have also provided a list of training curricula that teachers requested in order to meet the needs of students in their classrooms. However, there are limitations to this study: one of the limitations with using qualitative research methods is the reliance on self-report as a data source due to the possibility they create for reporter bias [67]. Self-reported accounts in qualitative research, however, are considered valuable and are the strength of this research paradigm. While bias is considered a problem within quantitative research due to the nature of this research being to prove a hypothesis, in qualitative research bias is less problematic as the nature of qualitative research is to provide a deeper understanding of a problem. Another limitation is the sample size of qualitative research, which limits generalizability. This study addressed this issue by recruiting a representative sample that included participants from a range of SES areas, gender, age, period of work, and the types of schools taught (Catholic, Independent and Public schools). Despite that, these results may not be generalisable, both in terms of the weighting given to experiences and the weighting given to teachers’ preference of training content. Indeed, while we did achieve saturation in the interview analysis, this study does not provide an exhaustive analysis of mental health training content. Nevertheless, further research, supported by larger sample, quantitative data sources, and data triangulation, may help identify further mental health curriculum development areas for teacher training. While our research is not intended to be conclusive, it does provide insight into further conversations about school-based mental health issues of students, and a platform on which to base future quantitative large-scale studies with a larger survey sample.

## Conclusion

The *Productivity Commission Report* found inconsistent approaches to pre-service teacher training and professional development in mental health and well-being. These initiatives failed to address the issues that impede schools from making a measurable difference in the mental health and well-being of children and adolescents in Australia. The present findings indicate the need for consistency across states and territories for child and adolescent mental health training in ITT and in-service teacher training. We have identified key areas for curriculum development. Teachers also asked for changes to school practices in managing the mental healthcare of students, specifically by increasing parent, community, and teacher engagement with healthcare professionals. This study finds that teacher training is urgently in need of revision to acknowledge the changing role of teachers globally in relation to the increasing mental health demands of students’ mental health needs in the classroom.

## Data Availability

The datasets used and/or analysed during the current study are available from the corresponding author upon reasonable request.
